# Acknowledgment of Reviewers

**DOI:** 10.1128/mBio.02302-17

**Published:** 2017-12-28

**Authors:** Arturo Casadevall

## EDITORIAL

On behalf of the editors of *mBio*, I gratefully acknowledge the following individuals, who served as reviewers for the journal in 2017. Their time and effort in reviewing articles have been essential in ensuring the high quality of our publications, and their help is greatly appreciated.

Robert B. Abramovitch
Luis A. Actis
Mark D. Adams
Michael W. W. Adams
Bruce Alberts
John F. Alcorn
Bree Aldridge
Gladys Alexandre
Francis Alonzo
Sam Alsford
Neal M. Alto
Amal Amer
Anne Anderson
Dan I. Andersson
David R. Andes
L. Aravind
Cesar A. Arias
Sandra K. Armstrong
Michelle M. Arnold
Gustavo Arrizabalaga
Louis Ates
Oliver Bader
Taeok Bae
Charles Baer
Mark Bailey
Scott Bailey
John F. Baines
Lauren O. Bakaletz
David A. Baker
Emily P. Balskus
Gert Bange
Daniel Barber
Joseph T. Barbieri
Amy Barczak
Debora E. Barnett Foster
Ralf Bartenschlager
Marek Basler
Pascale B. Beauregard
Dominik Begerow
Michael Behnke
Marcel A. Behr
Chase Beisel
Peter Belenky
George A. Belov
Gordon Morse Bennett
Andrew Bentley
Mary Berbee
Stephen Beres
Luiz E. Bermudez
Gurdyl Besra
Sébastien Besteiro
Bruce Beutler
Stephen M. Beverley
Ami S. Bhatt
Steven Biller
Oliver Billker
Gerald F. Bills
Russell E. Bishop
Julie Suzanne Biteen
Mike Blackman
Ira J. Blader
Scott Blanchard
Jorge Carlos Blanco
Lars Blank
Steven R. Blanke
James B. Bliska
Karl W. Boehme
Ivo G. Boneca
Seth Bordenstein
Alberto Bosque
Grant R. Bowman
John Dallas Boyce
Marc Bramkamp
Sara Branco
Alejandra Bravo
Patrick J. Brennan
Ariane Briegel
James Brien
Dustin Brisson
William J. Britt
Mathien Brochet
Michael A. Brockhurst
Nichole A. Broderick
C. Titus Brown
Daren W. Brown
Eric Brown
Eric D. Brown
Pamela J. B. Brown
Douglas F. Browning
Jeremy C. Brownlie
Harry Brumer
Nicolas Buchon
Carmen Buchrieser
Girbe Buist
Jürgen B. Bulitta
Saul Burdman
Briana M. Burton
Allen Buskirk
Mark J. Buttner
Jun Cai
Michael J. Calcutt
Charles H. Calisher
Andrew Camilli
Corey L. Campbell
Thomas Candela
Nicolas Carraro
Vern B. Carruthers
James E. Cassat
Roberto Cattaneo
Jean Celli
Jeremy Matthew Chacon
Debopam Chakrabarti
Mathias Chamaillard
Patricia Ann Champion
Josephine R. Chandler
Kartik Chandran
Wilfred Chen
Caroline Chenard
Leonid V. Chernomordik
Ambrose L. Cheung
Ludmila Chistoserdova
Chetan E. Chitnis
Byung-Kwan Cho
Sang-Nae Cho
Seemay Chou
Dennis Claessen
Cornelius J. Clancy
David J. Clarke
Ian N. Clarke
Thomas A. Clarke
Beatrice Clouet-D’Orval
Donald M. Coen
Tom Coenye
Taylor S. Cohen
Andre M. Comeau
Laurie E. Comstock
Andrew Conlan
Gregory M. Cook
Vaughn S. Cooper
Pierre Cornelis
Nicolas Corradi
Timothy L. Cover
Ben Cowling
Robert A. Cramer
Daniel Croll
Sean Crosson
Qiu Cui
Christina A. Cuomo
Anne M. Curtis
Michael Cynamon
Ross E. Dalbey
Ankur Dalia
Ajai A. Dandekar
Charles Darkoh
Kalyan Das
Alan R. Davidson
Mark R. Davies
Ian Christopher Davis
Scott C. Dawson
Christopher J. Day
Harry P. De Koning
Tania F. De Koning-Ward
Kirk W. Deitsch
Maurizio Del Poeta
Eric L. Delwart
Isabelle Derré
Andrea Dessen
Subhash Dhawan
Patricia I. Diaz
Victor J. DiRita
Yohei Doi
Leslie L. Domier
Delfina C. Dominguez
Tao G. Dong
Anna Dongari-Bagtzoglou
Maxwell Dow
Diana M. Downs
Michael Drebot
Arnold J. M. Driessen
Purnima Dubey
David Dubnau
Breck Duerkop
Christine Dunham
Julie C. Dunning Hotopp
Bruno Dupuy
Rebecca Ellis Dutch
Michelle Dziejman
Leo Eberl
Tassos Economou
Mira Edgerton
Joseph Edwards
Sabine Ehrt
Jonathan Eisen
Stéphane Emiliani
Alan Engelman
Luis Enjuanes
Slava Epstein
Clett Erridge
Manuel Espinosa
Matthew J. Evans
Robert Fagan
Daniel Falush
Ariberto Fassati
Mathias Faure
Karoline Faust
Michael J. Federle
Mario Feldman
Zongdi Feng
Scott Ferrenberg
Paul D. Fey
Sergio R. Filipe
Peter Charles Fineran
Utz Fischer
Matthew C. Fisher
David Fitzgerald
Ross Fitzgerald
David A. Fitzpatrick
Antje Flieger
Ervin Fodor
Donald N. Forthal
Jarrod R. Fortwendel
Kevin Foster
Timothy J. Foster
Betsy Foxman
Christopher A. Francis
Karen M. Frank
Michael J. Franklin
Sebastian Fraune
Eric O. Freed
Zachary Benjamin Freedman
W. Florian Fricke
Irwin Fridovich
Bettina C. Fries
Tobias Fuhrer
Kei Fujimura
Benjamin O. Fulton
Clay Fuqua
Richard L. Gallo
Denise A. Galloway
Michael G. Ganzle
Francisco García-Del Portillo
Adolfo García-Sastre
Gregory Gauthier
Ricardo T. Gazzinelli
Martin Gengenbacher
Dimitris Georgellis
Ralf Gerhard
Naama Geva-Zatorsky
Emma Mary Gibbin
Katherine E. Gibson
Jack A. Gilbert
Michael Gillings
Pierre Gladieux
Philippe Glaser
Sydney Glassman
Michael S. Glickman
Paul Goepfert
Daniel Goldberg
Ido Golding
William E. Goldman
Erin Goley
Mark Gomlesky
Ivo Gomperts Boneca
Jesus Gonzalo-Asensio
Vernita Gordon
Jeff Gore
Friedrich Götz
Richard L. Gourse
Elena A. Govorkova
Neil A. Gow
David C. Grainger
Jeffrey A. Gralnick
Lone Gram
Thomas Gramberg
Justin M. Greene
Nicole Grieshaber
Marc-Jan Gubbels
David Hackstadt
Bernard Hallet
Brian K. Hammer
Neal D. Hammer
Leendert W. Hamoen
Kaphoon Han
Yiping Han
William Hanage
Jo Handelsman
Scott Handley
Cara H. Haney
Lars Hestbjerg Hansen
Thomas E. Hanson
J. Marie Hardwick
Richard W. Hardy
Richard Harrigan
Steven Harris
Elizabeth L. Hartland
Aaron Hartmann
Graham F. Hatfull
Robert A. Heinzen
Antje Heit
John D. Helmann
Thomas Henry
Anat Herskovits
Christophe Herman
Volker Heussler
Mark J. Hickman
Penelope Higgs
Ann B. Hill
Deborah M. Hinton
Elizabeth B. Hirsch
Ya-Chi Ho
Monica Höfte
Deborah A. Hogan
Brenda G. Hogue
Edward C. Holmes
Stacy M. Horner
Malcolm J. Horsburgh
Alexander R. Horswill
Walid A. Houry
Mark Howarth
Guanghua Huang
Lori Huberman
Kelly T. Hughes
Jenni Hultman
Romney M. Humphries
Christopher Hunter
Aeron Christopher Hurt
Christopher Dwight Huston
Mark M. Huycke
Embriette R. Hyde
Iliyan Iliev
Jean-Luc Imler
Francesco Imperi
Javier Irazoqui
Ralph R. Isberg
Yoshikazu Ishii
Patrick Iversen
William T. Jackson
Jonathan Jacobs
William R. Jacobs, Jr.
Ilse D. Jacobsen
Anne Jamet
Amanda Jamieson
Urs Jenal
Haeyoung Jeong
Michael Joiner
Chris Jones
Stuart Jones
Joyce Jose
S. Joshi-Barve
Suckjoon Jun
Klaus Jürgens
Praveen Rao Juvvadi
Robert F. Kalejta
Alex J. Kallen
Sophien Kamoun
Petros C. Karakousis
Stephanie M. Karst
Fatah Kashanchi
Deepak Kaushal
Yoshihiro Kawaoka
Daniel B. Kearns
Frank Kempken
Melissa M. Kendall
Saleem A. Khan
Margaret Kielian
Kami Kim
Minsu Kim
Linda King
Natalia V. Kirienko
Karla Kirkegaard
Mareike Klinger-Strobel
Karl E. Klose
Leigh Knodler
Laura J. Knoll
Lester Kobzik
Rahul M. Kohli
Justin Kollman
Jay Kolls
Dennis L. Kolson
Roberto Kolter
Konstantinos T. Konstantinidis
Manfred Kopf
Konstantin V. Korotkov
Ákos T. Kovács
Karl-Heinz Krause
Barry N. Kreiswirth
Tino Krell
Eric J. Kremer
Anders Nils Kristoffersson
Lee Kroos
Karl Kuchler
Meta Kuehn
Jens H. Kuhn
Oscar P. Kuipers
Nirbhay Kumar
Sanjai Kumar
Andreas Kupz
Edo Kussell
Waldan Kwong
Borden Lacy
Frank Lafont
Richard J. Lamont
Tilman Lamparter
Morgan G. I. Langille
Gordon Langsley
Michael Lanzer
Luis F. Larrondo
Inigo Lasa
Jared Renton Leadbetter
Shin-Yi Lee Marzano
Benhur Lee
Chia Y. Lee
Jean Claire Lee
Seon-Woo Lee
Soo Chan Lee
Won-Jae Lee
Philippe Lemanceau
Jose Lemos
Debbie Lenshow
Francois Leuilier
Henry Levin
Stuart M. Levitz
Astrid Lewin
Susan Liebman
Honghuang Lin
Brett D. Lindenbach
Peter N. Lipke
Trevor Lithgow
George Liu
Volker Lohmann
Giovanni Longo
Susana López
Jose Juan Lopez-Rubio
Petra Louis
Andrew Lee Lovering
David A. Low
Steven K. Lower
Ting Lu
Yahai Lu
Martin Ludlow
Guangxiang George Luo
Joseph Lutkenhaus
Michael Lynch
Li-Jun Ma
Paul Magwene
Bernardo Alfredo Mainou
Nikhil S. Malvankar
Mark J. Mandel
Colin Manoil
Imma Margarit
William Margolin
Elisa Margolis
Luciano A. Marraffini
Mark Marsh
Michael Martin
Rowena Martin
Christopher J. Marx
Vega Masignani
Amy J. Mathers
Sohkichi Matsumoto
Jelle Matthijnssens
John McCauley
Kathleen McCoy
Bruce McDonald
James B. McKinlay
Patrik Medstrand
Jay Mellies
Roman Melnyk
Alita A. Miller
Joel Miller
Kiwamu Minamisawa
Nagendra N. Mishra
Aaron P. Mitchell
Thomas C. Mitchell
Tim Miyashiro
Christopher H. Mody
William W. Mohn
James W. B. Moir
Denis Molnar
Vanesa Mongelli
Cesare Montecucco
Ruth Montgomery
Frits R. Mooi
David M. Morens
Renato Morona
Juliet Morrison
Rafal Mostowy
Andrés Moya
Marie Muehe
Volker Mueller
Biswarup Mukhopadhyay
Daniel Muller
Matthew Mulvey
Carol A. Munro
Gerard Muyzer
Peter J. Myler
Raffael Nachbagauer
Carey Nadell
Peter Nagy
Moon H. Nahm
Michiko M. Nakano
Sjaak Neefjes
David E. Nelson
Jay Nelson
Graeme Nicol
Francisco Nicolás
Hiroshi Nikaido
Jacquin Niles
Juan Nogales
Minou Nowrousian
Jack H. Nunberg
Brian B. Oakley
Howard Ochman
Tim O’Connor
Audrey R. Odom
Marco Rinaldo Oggioni
Amanda G. Oglesby-Sherrouse
Anil Ojha
Kevin Olival
Antonio Oliver
Michal A. Olszewski
Eng Eong Ooi
Timothy J. Opperman
Marc Ostermeier
George O’Toole
Julie Overbaugh
Tim Overton
Daniel Pack
Mark S. Paget
Kelli L. Palmer
Tracy Palmer
Vijay Pancholi
Kai Papenfort
Rohinee Paranjpye
Tanya Parish
Vincent Parissi
John S. Parker
Sally R. Partridge
Marcela F. Pasetti
A. Lorena Passarelli
John T. Patton
Kabir Peay
Steen Pedersen
Richard M. Peek
Xuanxian Peng
Ines C. Pereira
John R. Perfect
Stanley Perlman
Steve Perlman
Everett Pesci
John W. Peters
Brook Peterson
William A. Petri, Jr.
Julie K. Pfeiffer
Andrew J. Phipps
Ted C. Pierson
Johann Pitout
Gregory Plano
Alexander Ploss
Laurent Poirel
Finn Pond
David L. Popham
Holly Popham
Veena Prahlad
B. V. Venkataram Prasad
Rajendra Prasad
Peter Punt
Lei Stanley Qi
David Queller
Ralf Rabus
Sylvain Raffaele
Manuela Raffatellu
Tracy Lyn Raivio
Sanjay Ram
Jay Rappaport
Chad A. Rappleye
David A. Rasko
Angela L. Rasmussen
Philip N. Rather
Pradipsinh K. Rathod
Adam J. Ratner
Julian Rayner
Matthew R. Redinbo
Michael Reese
Roland R. Regoes
Barbara Rehermann
Bernd H. Rehm
Chris R. Reisch
Carol Shoshkes Reiss
Larry Reitzer
Rolf Renne
Kelly C. Rice
Anthony R. Richardson
Bert K. Rima
Marjorie Robert-Guroff
Erle S. Robertson
D. Ashley Robinson
Nigel Robinson
Eduardo P. C. Rocha
Isabel Roditi
Matt B. Rogers
Eliora Z. Ron
Jason W. Rosch
David Rosen
Amy Rosenfeld
Amelia-Elena Rotaru
Sarah Rowland-Jones
David John Rowlands
Daniel Rozen
Gloria Rudenko
David Rudner
Bruce M. Russell
Julian Colin Rutherford
Alessandra Salvioli
Suryaprakash Sambhara
Dominique Sanglard
Dipali Sashital
Reetta Satokari
Karin Sauer
Claudio Scazzocchio
Luis M. Schang
Birgit E. Scharf
John Schiller
Klaus Schlaeppi
Patrick M. Schlievert
Patrick D. Schloss
Sarah Schmidt
Thomas Mitchell Schmidt
Monika Schmoll
Dirk Schnappinger
Olaf Schneewind
Jürgen Schneider-Schaulies
Karin Schnetz
Stephanie Schuller
Heide N. Schulz-Vogt
Olivier Schwartz
Stefan Schwarz
Martin Schwemmle
Petra Schwille
H. Steven Seifert
David A. Sela
Jeremy D. Semrau
Tae Seok Seo
Laura Serino
Ethan Settembre
Konstantin V. Severinov
Barbara Shacklett
William M. Shafer
Anand Shah
Jeffrey Shaman
Robert M. Q. Shanks
Aimee Shen
Donald C. Sheppard
Barbara Sherry
Pei-Yong Shi
Jessica Shiffman
Yu-Ling Shih
Thomas S. Shimizu
Sunny Shin
Kari Ann Shirey
Mark E. Shirtliff
Thomas J. Silhavy
Laurie Silva
Lynn L. Silver
Anthony P. Sinai
Steven Singer
Ramandeep Singh
Upinder Singh
Varsha Singh
Eric P. Skaar
Rebecca L. Skalsky
Robert Skov
David Skurnik
Emma Marie Caroline Slack
Richard A. Slayden
Jason G. Smith
Liz Sockett
Donald Sodora
Thierry Soldati
Greg A. Somerville
Justin Sonnenburg
Søren Johannes Sørensen
Carlos Yesid Soto Ospina
Philippe Soucaille
Victor Sourjik
Juliet V. Spencer
Tobias Spielmann
Michael Springer
Eric V. Stabb
Robert V. Stahelin
Thomas Stamminger
Jack T. Stapleton
Bärbel Stecher
Frank Stewart
Ann M. Stock
John Stolz
Paul D. Straight
Scott A. Strobel
Xin-Zhuan Su
Baolin Sun
Weina Sun
Yvonne Sun
Mehul Suthar
Richard E. Sutton
Elena Suvorova
Michiko E. Taga
Norio Takeshita
John Tam
Rita Tamayo
Pranita D. Tamma
Rick L. Tarleton
Christoph Tebbe
Luis Teixeira
Alice Telesnitsky
Martin Thanbichler
Volker Thiel
Anna D. Tischler
Andrew Tomaras
Matthew F. Traxler
Ralph A. Tripp
Bettina Tudzynski
M. F. Tuite
Kürsad Turgay
Keith Turner
Jane F. Turton
Anne-Catrin Uhlemann
Akhil B. Vaidya
Raphael Valdivia
Susana T. Valente
Laurence Van Melderen
Ed W. Van Niel
Douwe Van Sinderen
Jos A. G. Van Strijp
Daria Van Tyne
Brian C. Vanderven
Jan-Willem Veening
Jonathan L. Vennerstrom
Karen L. Visick
Waldemar Vollmer
Ekaterina Voronina
Romé Voulhoux
William G. Wade
Daniel Wall
Robert S. Wallis
Fiona Walsh
Timothy R. Walsh
David Wang
Guangyi Wang
Wei-Kung Wang
David W. Wareham
Matthew J. Wargo
Christopher M. Waters
Tilmann Weber
Michael R. Weigand
Jeffrey N. Weiser
David S. Weiss
Samantha Wellington
John Whitney
Brian Wigdahl
Daniel J. Wilson
Emma H. Wilson
Wade C. Winkler
Sebastian E. Winter
Matthew C. Wolfgang
Christiane Wolz
Patrick C. Y. Woo
Floyd L. Wormley, Jr.
Michael Worobey
Hui Wu
Liang Wu
De-Yu Xie
Chaoyang Xue
Hajime Yaegashi
Jinjuan Yan
Yajun Yan
Fitnat H. Yildiz
Kenichi Yokoyama
Kevin D. Young
Dong Yu
Sung-Hwan Yun
Thomas C. Zahrt
Michael Zasloff
Daniel R. Zeigler
Nikolay Zenkin
Jing-Ren Zhang
Jay Zhu
Jun Zhu
Xiaoping Zhu
Dan Zilberstein
Sara L. Zimmer
James E. A. Zlosnik
Peter Zuber

